# Adhesive *Bifidobacterium* Induced Changes in Cecal Microbiome Alleviated Constipation in Mice

**DOI:** 10.3389/fmicb.2019.01721

**Published:** 2019-08-13

**Authors:** Linlin Wang, Cailing Chen, Shumao Cui, Yuan-kun Lee, Gang Wang, Jianxin Zhao, Hao Zhang, Wei Chen

**Affiliations:** ^1^State Key Laboratory of Food Science and Technology, Jiangnan University, Wuxi, China; ^2^School of Food Science and Technology, Jiangnan University, Wuxi, China; ^3^International Joint Research Laboratory for Probiotics, Jiangnan University, Wuxi, China; ^4^Institute of Food Biotechnology, Jiangnan University, Yangzhou, China; ^5^Department of Microbiology and Immunology, National University of Singapore, Singapore, Singapore; ^6^National Engineering Research Center for Functional Food, Jiangnan University, Wuxi, China; ^7^Wuxi Translational Medicine Research Center and Jiangsu Translational Medicine Research Institute Wuxi Branch, Wuxi, China; ^8^Beijing Advanced Innovation Center for Food Nutrition and Human Health, Beijing Technology and Business University, Beijing, China

**Keywords:** *Bifidobacterium*, constipation, adhesion properties, SCFAs, gut microbiota

## Abstract

Constipation, which seriously affects living quality of people, is a common gastrointestinal disease. The engagement of the intestinal flora in the development of symptoms of constipation has been frequently hypothesized. In this study, constipated mice induced by loperamide were used to investige the alleviation of constipation by Bifidobacteria. Bifidobacteria was sorted out according to their adhesive properties into two groups. One group combined multiple strains of *Bifidobacterium* with adhesion property (CMB1), the other combined multiple strains of *Bifidobacterium* without adhesion property (CMB2). It was found that CMB1 can alleviate constipation more efficiently by improving the water, propionate and butyrate content in feces, and overall gastrointestinal transit time. Meanwhile, from the perspective of fecal microbiota, CMB1 alleviated constipation mainly by increasing the relative abundances of genera (*Bifidobacterium*, *Lactobacillus*, and *Prevotella*) associated with rapid bowel movement. From the perspective of cecal microbiota, CMB1 alleviated constipation mainly by increasing the relative abundances of genera *Lactobacillus*, *Bacteroides*, unclassified S24-7, *Dorea*, *Ruminococcus*, *Coprococcus*, and *Rikenella*, and decreasing the relative abundances of genera *Oscillospira*, *Odoribacter* and Unclassified F16, which are associated with methane production and colonic transit. Overall, changes of microbiota in caecum by CMB1 reflect the stage of constipation in mice more comprehensively than that in feces.

## Introduction

Usually, constipation is defined as infrequent or hard to pass bowel movements. It has a high incidence (2–36%) worldwide and complicated etiology (Rao et al., 2016). Its clinical symptoms include hard and dry feces, bowel movements that occur less than three times per week, rare occurrence of loose stools in the absence of laxatives and inadequate criteria for irritable bowel syndrome (Bharucha et al., 2013). In the past few years, dietary changes and the effects of physiological, psychological, sociological and other factors have led to an increase in the prevalence of constipation, which can severely affect people’s health and quality of life. Although the harmfulness of constipation is limited, it can be linked to increased risk of many related diseases such as Parkinson disease (Rossi et al., 2015) and colorectal cancer (Guérin et al., 2014). Therefore, it is very necessary to prevent and treat constipation. At present, the main drugs used to treat constipation are osmotic and secretory laxatives (Zhang et al., 2015). However, these therapies are susceptible to drug resistance to varying degrees and some lack efficacy. Many studies have shown that gut flora played a vital role in constipation. Constipation is related to the changes of gut microbiota, which may disturb cross-talk between the intestinal microflora, the intestinal endocrine system, the immune system and intestinal permeability (Caesar et al., 2015; Ge et al., 2017). Recent studies have revealed that constipation is related to imbalance in the intestinal microbiota, which mainly involve reduced levels of *Bifidobacterium* or *Lactobacillus* and an increase in pathogens (Chen et al., 2016). Supplementation with Bifidobacteria (Peter et al., 2006; Urita et al., 2015) or lactobacilli (Drouault et al., 2008; Williams et al., 2009; Yoon et al., 2015), either alone or combined, could prevent or treat constipation (Bu et al., 2007; Tabbers et al., 2011).

At present, many studies about the relationship between intestinal flora and diseases were based on fecal samples, which are easy to obtain but do not fully reflect the intestinal microbiota. In mice, microbiota in the caecum ferment carbohydrates that are unavailable in the small intestine. And it has been reported that there are significant differences in the structure of fecal, caecal and mucous membranes microbiota (Mao et al., 2016). Therefore, it is necessary to study the relationship between caecum content microbiota and constipation. In addition, our previous studies indicated that alleviation of constipation by *Bifidobacterium* might be related to the adhesion property of the strains (Wang et al., 2017a). It thus suggested that adhesion property may facilitate colonization of *Bifidobacterium* in the intestine. To date, there has been no reported research on the effect of adhesion property of *Bifidobacterium* on constipation. Therefore, it is of great significance to study the difference between fecal microbiota and caecum microbiota in mice after treated with Bifidobacteria with or without adhesion property.

In this study, constipated mice induced by loperamide were used to investigate the alleviation of constipation by Bifidobacteria. The Bifidobacteria were sorted by their adhesive property. It was found that strains with adhesion property can alleviate constipation more efficiently.

## Materials and Methods

### Reagents

Kits used to measure the levels of gastrointestinal (GI) neurotransmitters, motilin (MTL), gastrin (Gas), substance P (SP), endothelin (ET-1), somatostatin (SS) and vasoactive intestinal peptide (VIP) were purchased from Wen LE Bioengineering Institute (Shanghai, China).

Loperamide was dissolved in sterile water and its ultimate density was 1 mg/mL. For acticarbon solution, gum arabic 100 g and water 800 mL were boiled until the solution was transparent. Acticarbon 50 g was then added and boiled three times. After cooling, the solution was diluted with water to 1000 mL and stored at 4°C. Shake well before use.

### Bacteria Preparation

Ten *Bifidobacterium* strains from five species obtained from American Type Culture Collection (ATCC) or China General Microbiological Culture Collection (CGMCC) were stored at the Culture Collection of Food Microorganisms in Jiangnan University (CCFM, Wuxi, Jiangsu province, China). All the strains were cultured under anaerobic conditions for 24–48 h at 37°C in modified MRS (cMRS) broth supplemented with 0.05% w/v L-cysteine-HCl (Merck). To prepare active cultures for all experiments, all strains were consecutively reactivated in an anaerobic atmosphere at least three times using 3% (v/v) inoculum in cMRS broth at 37°C for 24–48 h before use. To use these strains in the animal experiments, the bacterial culture was centrifuged at 5000 × *g* for 10 min, washed twice with PBS, and centrifuged again to obtain the bacteria. The bacteria were divided into two groups (CMB1 and CMB2) of five based on their adhesion properties (Table 1). Adhesion properties of all these bacteria had been checked by cell adhesion assay *in vitro* and their adhesion characteristics can be found in our previous studies (Wang et al., 2017a). CMB1 refers to the multi-Bifidobacteria combination with the final concentration of 10^10^ CFU/mL by mixing all Bifidobacteria with adhesion properties at the same concentration. The preparation method of CMB2 was the same as that of CMB1.

**TABLE 1:** Bifidobacteria used in this study.

**Group**	**Strains**	**Source**	**Ways to acquire**	**Characteristics**
CMB1	*Bifidobacterium longum* CCFM 643	Human feces	Acquired CGMCC1.3048	All strains had adherence property.
	*Bifidobacterium breve* CCFM 670	Infant feces	Acquired CGMCC1.3013	
	*Bifidobacterium bifidum* CCFM 16	Infant feces	Acquired CGMCC1.3001	
	*Bifidobacterium adolescentis* CCFM 669	Human feces	Acquired ATCC15701	
	*Bifidobacterium animalis* CCFM 625	Mixed fermentation bacteria	Acquired ATCC15703	
CMB2	*Bifidobacterium longum* CCFM 642	Infant feces	Acquired ATCC15705	All strains had no adherence property.
	*Bifidobacterium breve* CCFM 622	Infant feces	Screened	
	*Bifidobacterium bifidum* CCFM 641	Infant feces	Acquired ATCC29521	
	*Bifidobacterium adolescentis* CCFM 626	Health human intestinal flora	Acquired CGMCC1.3003	
	*Bifidobacterium animalis* CCFM 624	Rabbit feces	Acquired CGMCC1.1852	

*CMB1, combining multiple strains of *Bifidobacterium* with adhesion property; CMB2, combining multiple strains of *Bifidobacterium* with no adherence property. CGMCC, China General Microbiological Culture Collection; ATCC, American Type Culture Collection.*

### Experimental Design

Eight-week-old male BALB/c mice were obtained from Shanghai Laboratory Animal Center (Shanghai, China). The mice were kept in polyvinyl chloride (PVC) cages under environmentally controlled conditions with a 12-h light-dark cycle and standard commercial mouse feed and water were provided *ad libitum*. This study was approved by the Ethics Committee of the Jiangnan University, China (JN. No. 20150326-0110-21) and performed at the Experimental Animal Center of the Jiangnan University [License No. SYXK(SU)2016-0045].

To examine the preventive effects of CMB on constipation, 32 mice were used after a week-long adaptive period. The mice were randomly separated into four groups (*n* = 8): normal (healthy mice), control (constipation mice without treatment), CMB1 (constipation mice treated with CMB with adhesion properties) and CMB2 (constipation mice treated with CMB with no adhesion properties).

The mice were fasted overnight before the first experiment. The normal and control groups were given 0.25 mL normal saline (NS) using intragastric administration once a day for 17 days. The CMB1 and CMB2 groups were intragastrically administered 0.25 mL of normal saline solution containing 4 × 10^10^ CFU/mL CMB1 or CMB2, respectively, daily for 2 weeks. All of the groups, except the normal group, were given loperamide (0.25 mL) intragastrically from day 15 to day 17 to induce constipation (Qian et al., 2013). Changes in food intake, water intake, and body weight were measured once per day at 9:00 AM throughout the experimental period. On the last day of the experiment, the mice were anesthetized by dipping ether with cotton in a relatively sealed space. The mouse beard was cut with surgical scissors to prevent hemolysis. Fixed the mouse, used the tweezers to clamp the eyeball, let the blood flow vertically into the centrifuge tube, put the centrifuge tube at room temperature for 2 h and then put it into the 4°C refrigerator for 3 h and centrifuged at 3000 × *g* for 15 min to obtain serum. The abdomen was opened, the cecum of the mouse was cut with a surgical scissors and the contents of the cecum were aspirated into the EP tube using a syringe without a needle, then gently put the colon in sterile saline to remove the remaining contents, and finally placed the colon into the EP tube and stored at −80°C until analysis. All operations were performed under sterile conditions. The experimental design is shown in Supplementary Figure S1.

### Detection of Constipation-Related Indices

The relative indices of constipation (i.e., the water content of the feces, the small intestinal transit rate and the first black stool defecation time), stool collection and SCFAs in feces were measured as previously described (Wang et al., 2017a). Feces were collected every three days and stored at −80°C. The water content of the feces was measured by the difference between the wet and dry weights of the feces, the small intestinal transit rate was evaluated by the distance traveled by an acticarbon solution relative to the overall length of the small intestine and the first black stool defecation time was measured by the time between the gavage of acticarbon solution and the appearance of darkened feces.

### Detection of the GI Neurotransmitters Levels in Serum

The GI neurotransmitters levels in the serum were determined by an enzyme-linked immunosorbent assay (ELISA) instrument according to the manufacturer’s instructions (Microplate Spectrophotometer Multiskan Go, Thermo Scientific, Waltham, MA, United States). The experiments mainly include the preparation of standard curve and the detection of sample absorption value (OD450) according to the standard curve in units of ng/L.

### 16S rDNA Sequencing and Bioinformatics Analysis

The enteric microorganisms in the fecal and caecal samples were measured using a metagenomics method (Wang et al., 2017a). Microbial genomic DNA was obtained using a FastDNA Spin Kit for Soil (MP Biomedical). The V4 region of the 16S rDNA was amplified by PCR. The products were purified and quantified using Gene Clean Turbo (MP Biomedical) and the Quant-iT PicoGreen dsDNA Assay Kit (Life Technologies), respectively. Libraries were prepared using TruSeq DNA LT Sample Preparation Kits (Illumina) and sequenced by Illumina MiSeq using the MiSeq Reagent Kit.

The QIIME pipeline was used to analyze the 16S rDNA sequence data (Caporaso et al., 2010). The raw sequences were screened. The short lengths (<200 bp) were then removed, and the pair-end reads that overlapped longer than 10 bp and without any mismatch were assembled according to their overlap sequence. The sequences were then clustered into operational taxonomic units (OTUs) based on 97% identity using QIIME^1^. The representative sequences for each OTU were aligned to identify the species using PyNAST in QIIME. Rarefaction curves for alpha diversity were generated to assess the efficiency of the sequencing depth for representing and comparing microbial communities. Species richness was estimated using Chao-1 (Faith and Baker, 2006). The beta diversity of the microbial communities was determined by visual assessment using principle coordinate analysis (PCoA) plots and by an analysis of similarity calculated based on weighted UniFrac distances (QIIME) according to one-way non-parametric multivariate analysis of variance.

### Statistical Analysis

The data were presented as mean ± SD and analyzed using GraphPad Prism 5 and Origin 8.5. The differences between the samples were analyzed by one-way ANOVA with Duncan’s multiple range test. The results were considered significant when *p* < 0.05.

## Results

### *Bifidobacterium* Combination With Adhesion Properties Significantly Improved the Symptoms of Constipation

**FIGURE 1:**
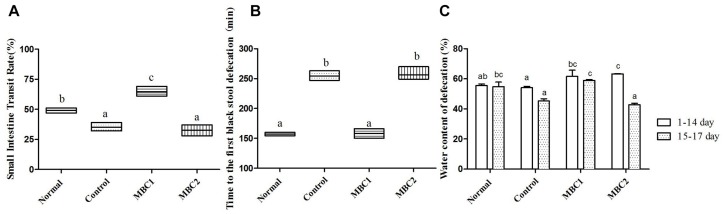
Defecation status of mice during the experiments. **(A)** Small intestinal transit rate; **(B)** Time to the first black stool defecation; **(C)** water content of defecation; a–c, mean values with different letters over the bars are significantly different (*p* < 0.05) according to Duncan’s multiple range test.

Before constipation was induced, the water content of the feces was higher in CMB1, and CMB2 groups than in the control and normal groups (Figure 1C, *p* < 0.05). With the intake of loperamide, the fecal water content showed a downward trend in all groups, which indicated that loperamide induced constipation in mice.

Compared with the constipation group, the symptoms of constipation (i.e., the water content of the feces, the small intestinal transit rate and the first black stool defecation time) were significantly improved in CMB1 group (Figure 1, *p* < 0.05). Meanwhile, there was no statistical difference between the CMB2 and control groups. These results demonstrate that CMB2 had no effect on constipation induced by loperamide. There was no significant difference in weight gain, feed and water consumption between the mice throughout the experiment. This indicated that constipation had no effect on the weight and appetite of mice.

### *Bifidobacterium* Combination With Adhesion Properties Significantly Improved the GI Neurotransmitters Levels in Serum

**FIGURE 2:**
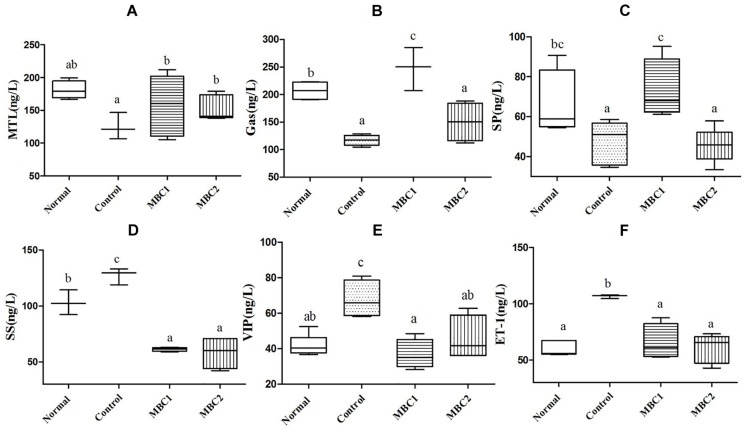
Effects of CMB on constipation-related GI neurotransmitters. **A,** MTL; **B,** Gas; **C,** SP; **D,** SS; **E,** VIP; **F,** ET-1; a–c, mean values with different letters over the bars are significantly different (*p* < 0.05) according to Duncan’s multiple range test.

Constipation-related neurotransmitters, such as MTL, Gas, SP, ET-1, SS and VIP, play an important role in regulating gastrointestinal motility. In addition, the secretion of GI neurotransmitters is different in disease and normal state. Therefore, we studied these GI neurotransmitters in serum of mice after treated with CMB. The results showed that CMB1 obviously increased the levels of MTL, Gas and SP and decreased the levels of SS, VIP and ET-1. The variation tendency of GI neurotransmitters in CMB2 group were the same as that in CMB1 group, except for Gas and SP (Figure 2, *p* < 0.05). In CMB1-treated group, the Bifidobacteria combination showed preferable effect in relieving constipation. These results indicated that the difference in relieving constipation between CMB1 and CMB2 may be related to the levels of Gas and SP in serum.

### *Bifidobacterium* Combination With Adhesion Properties Significantly Raised the Concentration of SCFAs in Fecal Samples

The contents of each SCFA in the feces are shown in Tables 2, 3. Before constipation, acetate, propionate and butyrate levels were fairly steady in normal and constipation control groups, while the concentrations of acetate and total acids increased, and the level of butyrate decreased in CMB1 and CMB2 groups. After the induction of constipation, the concentrations of acetate showed the same level in normal and control groups. Only propionic, butyric and total acids decreased compared to normal group. In addition, acetic, propionic and butyric acid concentrations increased in CMB1 group, whereas there was no significant difference between control and CMB2 groups in the concentrations of propionic and butyric acids. These results indicated that increasing the concentration of propionic and butyric acids in feces might be associated with the relief of constipation symptoms.

**TABLE 2:** SCFAs in feces before constipation.

**SCFAs, μmol/g**	**Normal**	**Control**	**CMB1**	**CMB2**
Acetic acid	13.71 ± 1.33^a^	13.32 ± 2.19^a^	24.62 ± 0.67^b^	28.59 ± 0.3^c^
Propionic acid	2.25 ± 0.14^a^	1.98 ± 0.3^a^	2.38 ± 0.25^a^	2.24 ± 0.19^a^
Butyric acid	5 ± 0.11^c^	4.51 ± 0.4^bc^	3.62 ± 0.16^a^	4.42 ± 0.36^b^
Total acid	20.95 ± 1.57^a^	19.72 ± 2.11^a^	30.62 ± 0.99^b^	35.34 ± 0.86^c^

*CMB1 group: (combining multiple strains of *Bifidobacterium* with adhesion property), 1 × 10^10^ CFU; CMB 2 group: (combining multiple strains of *Bifidobacterium* with no adhesion property), 1 × 10^10^ CFU. ^*a*–*c*^ Mean values with different letters over bars are significantly different (*p* < 0.05) according to Duncan’s multiple range test.*

**TABLE 3:** SCFAs in feces after constipation.

**SCFAs, μmol/g**	**Normal**	**Control**	**CMB1**	**CMB2**
Acetic acid	13.85 ± 1.15^a^	12.57 ± 0.43^a^	35.9 ± 2.29^c^	20.45 ± 0.12^b^
Propionic acid	2.20 ± 0.19^b^	1.68 ± 0.3^a^	3.37 ± 0.28^c^	1.88 ± 0.11^ab^
Butyric acid	4.69 ± 0.66^b^	1.67 ± 0.32^a^	4.46 ± 0.36^b^	1.63 ± 0.25^a^
Total acid	20.74 ± 1.71^b^	15.92 ± 0.80^a^	43.73 ± 2.76^c^	23.96 ± 0.38^b^

*CMB1 group: (combining multiple strains of *Bifidobacterium* with adhesion property), 1 × 10^10^ CFU; CMB2 group: (combining multiple strains of *Bifidobacterium* with no adhesion property), 1 × 10^10^ CFU; ^*a*–*c*^ Mean values with different letters over bars are significantly different (*p* < 0.05) according to Duncan’s multiple range test.*

### Adhesive *Bifidobacterium* Combination Affected Fecal Microbiota in Different Way to Non-adherent *Bifidobacterium* Combination

To evaluate the influences of the CMB on the intestinal flora, we analyzed the fecal samples of healthy and constipated BALB/c mice gavaged with CMB1 and CMB2 for 17 days. A dataset containing 325,440 high-quality sortable 16S rDNA gene orders was produced from 32 fecal specimens using MiSeq sequencing. The average sequence read was 10,170 per sample. Representative sequences of all of the sequences were clustered, and a 97% sequence similarity cut-off was used. The number of OTUs per sample ranged between 433 and 1952. We performed association tests based on α- and β- diversity measures. There was a remarkable difference in α-diversity between the normal and constipation control groups (Supplementary Figure S2). After induced constipation by loperamide, the α-diversity indices (Chao-1, observed species and phylogenetic diversity) shown a sharp declined in constipation control and CMB2 groups. In contrast, CMB1 obviously increased the α-diversity indices. It indicated that CMB1 significantly increased the taxa richness of fecal microbiota, while CMB2 had no effect on the decrease of fecal microbial diversity caused by constipation.

The β-diversity of the fecal flora in mice given CMB1 and CMB2 was revealed by using unweighted UniFrac matrixes. As shown in Supplementary Figure S3, the light blue symbol represents the normal group and was located at the lower section of the PCA diagram, while the red symbol represents the constipation control group and was shift from the lower section of the score plot to the left, indicating that constipation changed the fecal microbiota structure of mice. The green and dark blue symbols, respectively, represent the CMB1 and CMB2 groups and were, respectively, located at the upper middle and upper right section of the PCA diagram, indicating that CMB significantly changed the fecal microbiota structure of mice. However, CMB1 and CMB2 affected the fecal microbiota through absolutely different ways.

**FIGURE 3:**
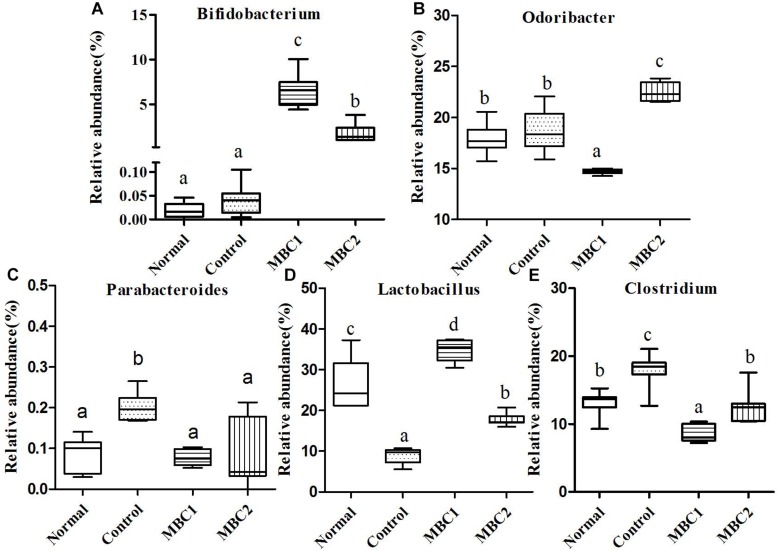
Relative abundance (%) of fecal microbial taxa at the genus level in constipated mice fed CMB. **(A)** *Bifidobacterium*, **(B)** *Odoribacter*, **(C)** *Parabacteroides*, **(D)** *Lactobacillus*, and **(E)** *Clostridium*.

**FIGURE 4:**
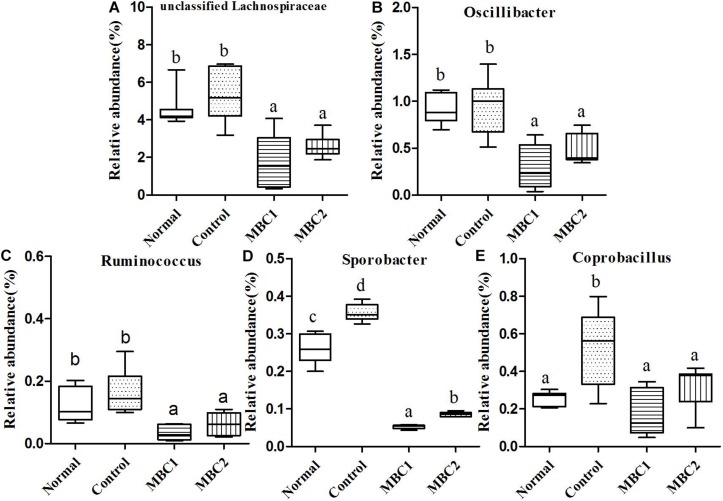
Relative abundance (%) of fecal microbial taxa at the genus level in constipated mice fed CMB. **(A)** Unclassified Lachnospiraceae, **(B)** *Odoribacter*, **(C)** *Ruminococcus*), **(D)** *Sporobacter*, and **(E)** *Coprobacillus*.

**FIGURE 5:**
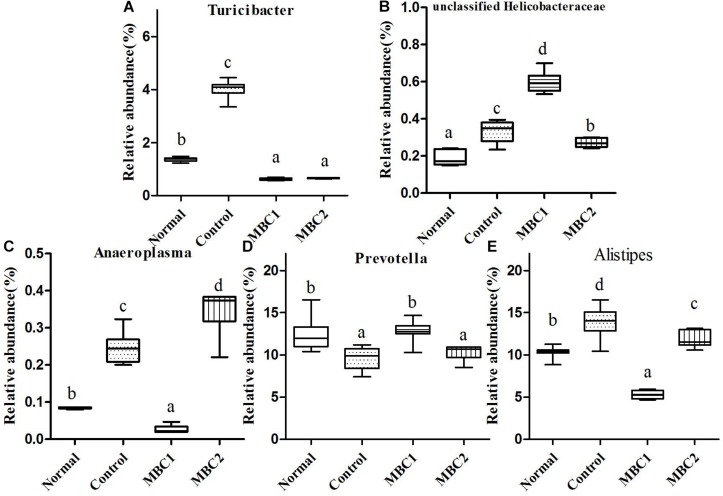
Relative abundance (%) of fecal microbial taxa at the genus level in constipated mice fed CMB. **(A)** *Turicibacter*, **(B)** unclassified Helicobacteraceae, **(C)** *Anaeroplasma*, **(D)** *Prevotella* and **(E)** *Alistipes*.

A total of 30,627 sequences were appointed to 1,952 OTUs, which agglomerated into 41 genera and 8 phyla using the Ribosomal Database Project (RDP) classifier. Changes in the taxa that were obviously different between treatments are shown in Figures 3–5.

#### Actinobacteria

Two genera (*Bifidobacterium* and *Adlercreutzia*) were included in this phylum. At the genus level, the level of *Bifidobacterium* in CMB groups were significantly higher than in constipation group. There was no significant difference among groups in the level of *Adlercreutzia* (Figrue 3, *Bifidobacterium*).

#### Bacteroidetes

Seven genera *Bacteroides*, *Odoribacter*, *Parabacteroides*, *Barnesiella*, *Rikenella*, *Prevotella* and *Alistipes* were included in this phylum. *Odoribacter* was the lowest in CMB1 group, followed by normal and control groups and then CMB2 group. The relative abundance of *Parabacteroides* decreased to normal level after treated with CMB. The relative abundance of *Prevotella* recovered to normal level in CMB1 treatment group, whereas there was no significant difference between CMB2 and constipation control group. *Alistipes* was the lowest in CMB1 treatment group and the highest in constipation control group. There was no significant difference among groups in the level of other genera (Figrues 3, 5, *Odoribacter*, *Parabacteroides*, *Prevotella* and *Alistipes*).

#### Deferribacteres

This phylum included a single genus, *Mucispirillum*. There was no significant difference among groups in the level of *Mucispirillum*.

#### Firmicutes

Twenty-seven genera (such as *Staphylococcus*, *Lactobacillus*, *Lactococcus*, *Streptococcus*, *Clostridium*, *Eubacterium*, *Blautia*, *Dorea*, *Oscillibacter*, *Ruminococcus*, *Sporobacter*, *Coprobacillus*, *Turicibacter* and so on) were included in this phylum. At the genus level, the relative abundance of unclassified Lachnospiraceae, *Clostridium*, *Sporobacter*, *Coprobacillus*, *Turicibacter*, *Oscillibacter* and *Ruminococcus* decreased significantly in CMB groups compared to control group. Meanwhile, the relative abundance of *Clostridium* and *Sporobacter* were higher in CMB2 treatment group than that in CMB1 group. In contrast, the relative abundance of *Lactobacillus* was the highest in CMB1 treatment group, followed by normal and CMB2 groups and then the constipation control group (Figures 3–5). There was no significant difference among groups in the level of other genera.

#### Proteobacteria

Only one genus was found in this phylum. The relative abundance of unclassified Helicobacteraceae only increased in CMB1 treatment group, whereas there was no significant difference among other groups (Figure 5).

#### TM7

This phylum contained one genus, the unclassified TM7. There was no significant difference among groups in this genus.

#### Tenericutes

*Anaeroplasma* was the only genus found in this phylum. Its level was highest in CMB2 group followed by the control and normal groups and then the CMB1 group (Figure 5, *Anaeroplasma*).

#### Verrucomicrobia

*Akkermansia* was the only genus found in this phylum. Its level was only increased in CMB1 treatment group, whereas there was no significant difference among other groups.

In brief, in CMB1-treated group, the relative abundances of *Bifidobacterium*, *Lactobacillus*, *Akkermansia*, *Prevotella* and unclassified Helicobacteraceae significantly increased and the levels of *Clostridium* and *Anaeroplasma* in feces samples decreased compared to CMB2-treated group. Moreover, the levels of *Bifidobacterium*, *Lactobacillus*, *Akkermansia* and *Prevotella* were negatively correlated with constipation, while *Clostridium* and *Anaeroplasma* were positively correlated with constipation. Therefore, after CMB1 intervention, the gut microbiota in constipated mice tended to be beneficial to host health.

### Adhesive *Bifidobacterium* Combination Affected Caecal Microbiota in Different Way to Non-adherent *Bifidobacterium* Combination

A dataset containing 373,216 high-quality sortable 16S rDNA gene orders was produced from 32 fecal specimens using MiSeq sequencing. The average sequence read was 11,663 per sample. Representative sequences of all of the sequences were clustered, and a 97% sequence similarity cut-off was used. The number of OTUs per sample ranged between 412 and 1330. We performed association tests based on α- and β- diversity measures. The normal and constipation control groups had obvious differences in α- diversity based on species richness, the observed species and diversity (Supplementary Figure S4). After CMB treatment, the chao-1 index and observed species showed significant changes in CMB1 group. The phylogenetic diversity index was obviously changed in CMB1 and CMB2 groups compared with the constipation group. The β diversity of the caecal microbiota was assessed using unweighted UniFrac matrixes. As shown in Supplementary Figure S5, constipation changed the caecal microbiota structure of mice greatly, while CMB significantly changed the caecal microbiota structure of mice. Different from the absolutely different ways by CMB1 and CMB2, respectively, these two *Bifidobacterium* combinations showed a certain overlap in the caecal microbiota regulation.

**FIGURE 6:**
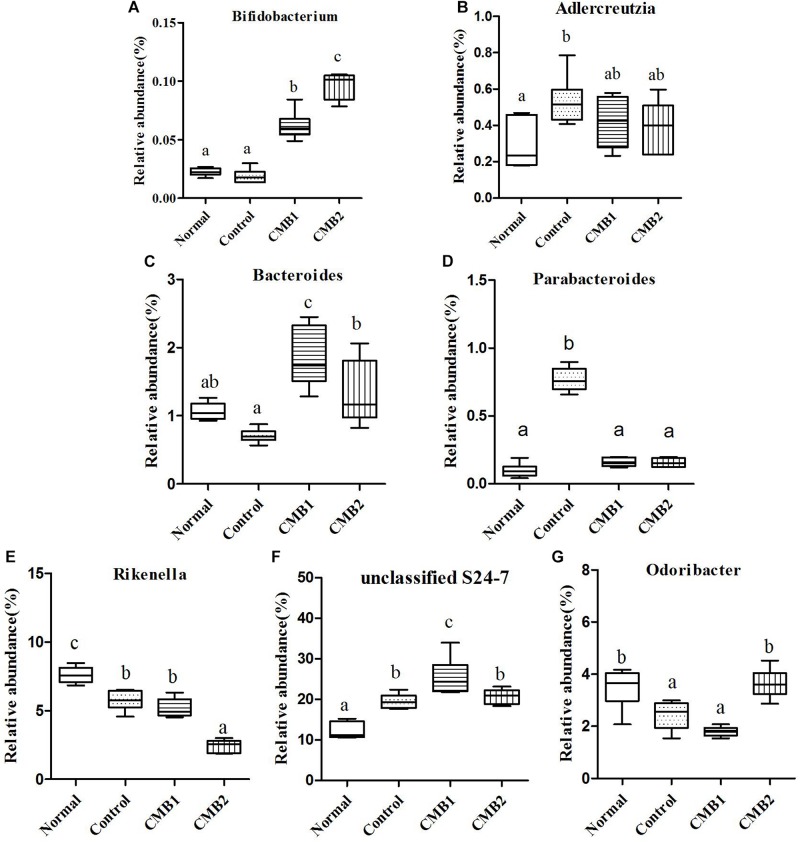
Relative abundance (%) of caecal microbial taxa at the genus level in constipated mice fed CMB. **(A)** *Bifidobacterium*, **(B)** *Adlercreutzia*, **(C)** *Bacteroides*, **(D)** *Parabacteroides*, **(E)** *Rikenalla*, **(F)** Unclassified S24-7, and **(G)** *Odoribacter*.

**FIGURE 7:**
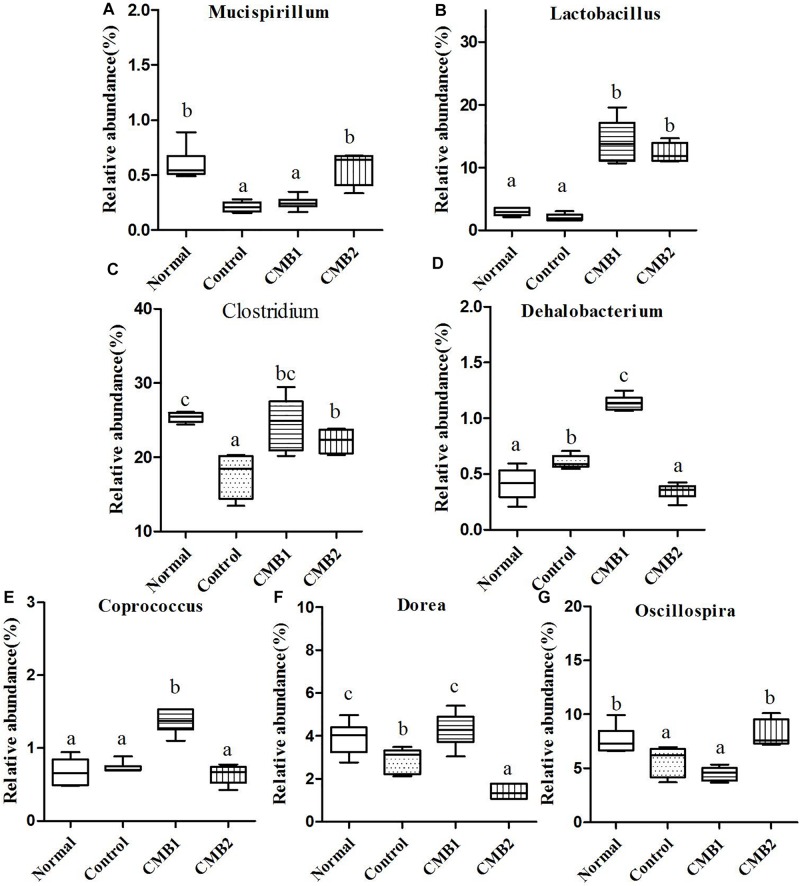
Relative abundance (%) of caecal microbial taxa at the genus level in constipated mice fed CMB. **(A)** *Mucispirillum*, **(B)** *Lactobacillus*, **(C)** *Clostridium*, **(D)** *Dehalobacterium*, **(E)** *Coprococcus*, **(F)** *Dorea*, and **(G)** *Oscillospira*.

**FIGURE 8:**
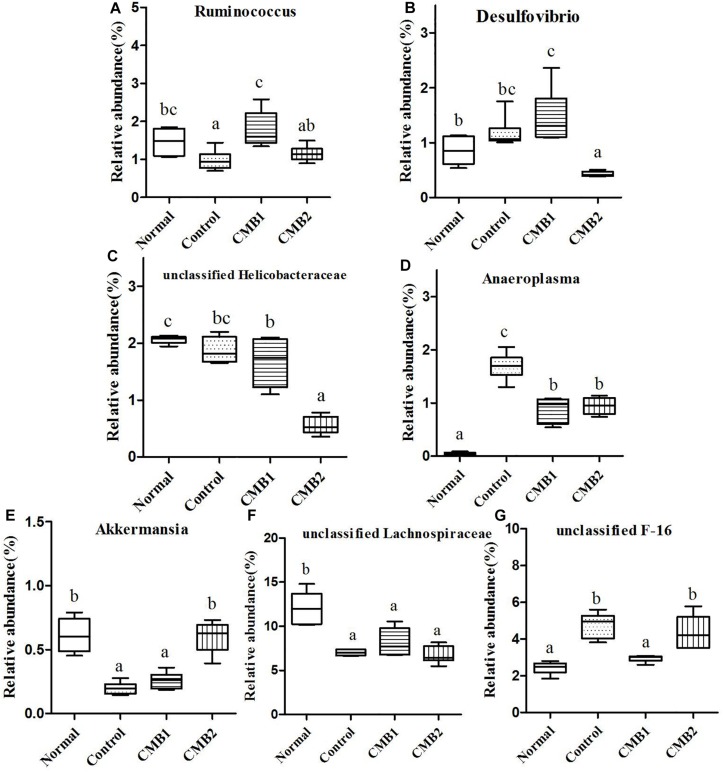
Relative abundance (%) of caecal microbial taxa at the genus level in constipated mice fed CMB. **(A)** *Ruminococcus*, **(B)** *Desulfovibrio*, **(C)** unclassified Helicobacteraceae, **(D)** *Anaeroplasma*, **(E)** *Akkermansia*, **(F)** unclassified Lachnospiraceae, and **(G)** unclassified F-16.

A total of 29,193 sequences were assigned to 1,396 OTUs that were clustered into 54 genera and 9 phyla using the RDP classifier. Changes in taxa are shown in Figures 6–8.

#### Actinobacteria

Two genera were found in this phylum, *Adlercreutzia* only increased in control group, whereas there was no significant difference among other groups. The relative abundance of *Bifidobacterium* was the highest in CMB2 treatment group, followed by CMB1 treatment group and then normal and control groups (Figure 6, *Bifidobacterium*).

#### Bacteroidetes

Twelve genera were found in this phylum. At the genus level, the level of *Bacteroides* was the highest in CMB1 treatment group, followed by CMB2 and then the normal and control groups. The levels of *Parabacteroides* only increased significantly in control group, whereas there was no significant difference among other groups. The level of *Rikenella* was the highest in CMB1 group and the lowest in CMB2 treatment group, whereas there was no significant difference between normal and control group. The relative abundance of unclassified S24-7 was the highest in CMB1 treatment group, followed by CMB2 and control groups and then the normal group. The relative abundance of *Odoribacter* recovered to normal level after treated with CMB2, whereas there was no significant difference between CMB1 and control group. There was no significant difference among groups in the level of other genera (Figure 6).

#### Cyanobacteria

Unclassified YS2 was the only genus found in this phylum, and there was no significant difference among groups in this genus.

#### Deferribacteres

*Mucispirillum* was the only genus found in this phylum, and the relative abundance of *Mucispirillum* recovered to normal level after treated with CMB2, whereas there was no significant difference between CMB1 and control group (Figure 7).

#### Firmicutes

Twenty-six genera (such as *Lactobacillus*, unclassified Clostridiales, *Clostridium*, *Dehalobacterium*, unclassified Lachnospiraceae, *Coprococcus*, *Dorea*, unclassified Ruminococcaceae, *Oscillospira*, *Ruminococcus* and so on) were found in this phylum. The abundances of *Dehalobacterium*, *Dorea*, *Coprococcus*, and *Ruminococcus* were significantly higher in CMB1group compared to CMB2 group, whereas the abundance of *Oscillospira* was obviously lower in CMB1group compared to CMB2 group. The relative abundance of *Lactobacillus*, and *Clostridium* increased significantly in CMB groups compared to control group. The relative abundance of unclassified Lachnospiraceae decreased significantly in all treatment groups, except the normal group. And there was no significant difference among groups in the level of other genera (Figures 7, 8).

#### Proteobacteria

Seven genera were found in this phylum. The level of *Desulfovibrio* was the highest in CMB1 group and the lowest in CMB2 treatment group, whereas there was no significant difference between other groups. Meanwhile, the level of unclassified Helicobacteraceae was the lowest in CMB2 treatment group, and the highest in normal group, whereas there was no significant difference between control and CMB2 treatment group. There was no significant difference among groups in the level of other genera (Figures 7, 8).

#### TM7

This phylum included one genus, unclassified F16. Its level was decreased to normal group in CMB1 treatment group, whereas there was no significant difference between CMB2 and control group (Figure 8).

#### Tenericutes

This phylum included one genus. The relative abundance of *Anaeroplasma* was the highest in control group, followed by CMB treatment groups and then the normal group (Figure 8).

#### Verrucomicrobia

*Akkermansia* was the only genus found in this phylum. Its level was only increased in CMB treatment group, whereas there was no significant difference between normal and control group (Figure 8).

In brief, after CMB intervention, the common effect of CMB1 and CMB2 on caecum microbiota was that they significantly elevated the relative abundances of *Lactobacillus*, *Clostridium*, *Akkermansia* and *Bifidobacterium* and reduced the relative abundances of *Anaeroplasma*. The difference was that in CMB1-treated group, the relative abundances of *Bacteroides*, *Rikenella*, unclassified S24-7, *Dorea*, *Coprococcus, Ruminococcus*, *Dehalobacterium* and *Desulfovibrio* significantly increased and the relative abundances of *Oscillospira, Odoribacter, Mucispirillum* and unclassified F16 decreased, while all these changes were opposite in CMB2-treated group. Considering the result that CMB1 alleviated constipation effectively, it seemed that the differences on cecum microbiota between CMB1- and CMB2- treated groups might be closely related to the situation of constipation in mice.

**FIGURE 9:**
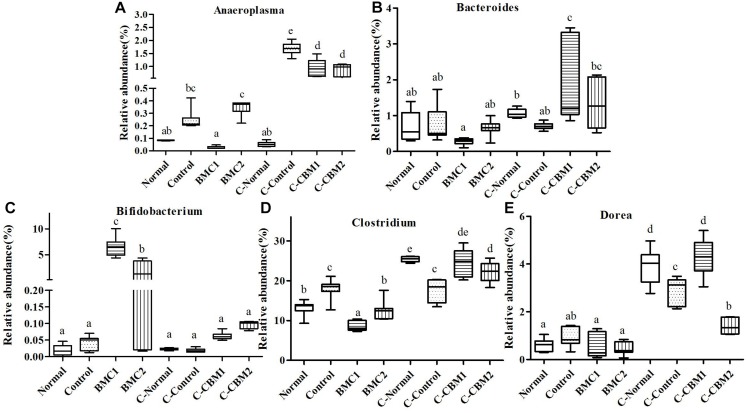
The genus-level comparison of fecal samples and caecum content samples. **(A)** *Anaeroplasma*, **(B)** *Bacteroides*, **(C)** *Bifidobacterium*, **(D)** *Clostridium*, and **(E)** *Dorea*.

**FIGURE 10:**
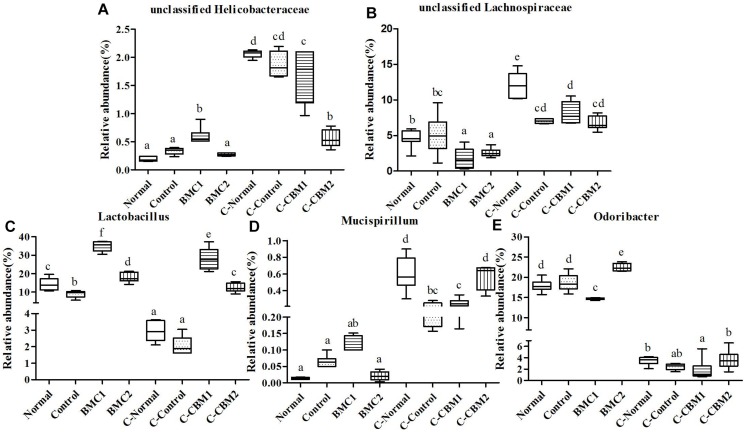
The genus-level comparison of fecal samples and caecum content samples. **(A)** Unclassified Helicobacteraceae, **(B)** unclassified Lachnospiraceae, **(C)** *Lactobacillus*, **(D)** *Mucispirillum*, and **(E)** *Odoribacter*.

**FIGURE 11:**
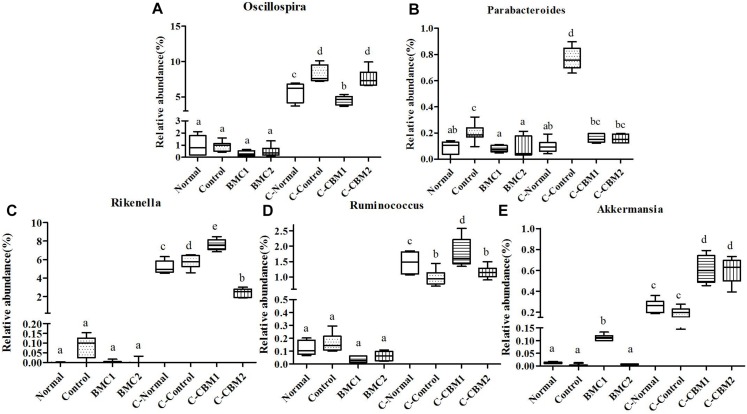
The genus-level comparison of fecal samples and caecum content samples. **(A)** *Oscillospira*, **(B)** *Parabacteroides*, **(C)** *Rikenella*, **(D)** *Ruminococcus*, and **(E)** *Akkermansia*.

Comparing the fecal flora with the cecum content flora at the genus level, we found that there were 15 genera with significant differences between these two samples. As shown in Supplementary Figure S6. the fecal microbiota was located at the right of the score plot, whereas the caecal microbiota was located at the left of the score plot. It indicated that there were significant differences between fecal microbiota and caecum microbiota. Differences in taxa are shown in Figures 9–11.

At the genus level, there were 15 genera with significant differences between fecal microbiota and caecum microbiota, and 11 of these 15 genera showed that the relative abundance of caecum samples was significantly higher than that of fecal samples. In contrary, the relative abundances of *Lactobacillus*, *Bifidobacterium*, and *Odoribacter* were higher in fecal samples than in cecum samples (Figures 9, 10). In addition, the relative abundances of *Bacteroides* and *Clostridium* in feces sample and caecum sample presented the opposite tendency in CMB1 group, whereas the level of *Anaeroplasma* showed the same tendency (Figure 9). The relative abundances of *Dorea*, *Rikenella* and *Ruminococcus* in cecum sample increased obviously in CMB1-treated group, whereas the level of *Oscillospira* in cecum sample decreased obviously in CMB1-treated group. And the level of *Odoribacter* in fecal sample decreased obviously in CMB1-treated group (Figures 9–11).

## Discussion

Although many efforts have been made to treat constipation, it remains one of the most common diseases worldwide. Therefore, it is urgent to explore an effective, safe and low-toxicity methods to treat constipation. Clinical studies have found that gut microbiota plays an important role in the occurrence and development of constipation. Thus, micro-ecological therapy has gradually replaced the traditional treatment for constipation.

A large number of animal and human experiments have confirmed that probiotics can alleviate constipation (Bu et al., 2007; Tabbers et al., 2011; Urita et al., 2015). However, the adhesion property of strains is the premise of this study in evaluating the probiotic effect of strains. Therefore, in this study, ten strains of Bifidobacteria were divided into two groups according to their adhesion property to investigate their efficacy in alleviating constipation. The results showed that CMB1 (combination of the adhesive Bifidobacteria) significantly improved the water content of feces, the small intestinal transit rate and the first black stool defecation time, whereas the CMB2 (combination of non-adhesive Bifidobacteria) had no effect on constipation. This result was consistent with our previous study (Wang et al., 2017a). The results showed that whether a single strain or multiple strains of *Bifidobacterium* are used, the effect of relieving constipation is related to the adhesion property of the bacteria.

The known factors affecting intestinal motility are nervous systems (intestinal nervous system and autonomic nervous system), immune system, intestinal microbiota and their metabolites. Any disorder or dysfunction in any of these factors can lead to intestinal motility disorders (constipation or diarrhea). Therefore, in this study, GI neurotransmitters levels were measured to determine the effect of Bifidobacteria on constipation-related intestinal nervous activities. The results showed that the reduced GI neurotransmitters in serum of constipation mice tended to be up-regulated in CMB1 intervention group, while CMB2 intervention had no effect on recovery of neurotransmitters (MTL, Gas and SP). Thus, CMB1 may alleviate constipation by up-regulating neurotransmitters (MTL, Gas and SP). Considering the adhesion properties of *Bifidobacterium*, we believe that the bacteria with adhesion property may have a greater chance of establishing close contact with and colonizing the intestine to exert GI neurotransmitter regulatory effects.

It has been suggested that the changes of gut flora and metabolites may be the important reasons for the pathophysiological changes in constipation. Several previous studies have reported that an increased concentration of SCFAs in the intestine is beneficial to constipation (Salminen and Salminen, 1997; Veiga et al., 2014). SCFAs are produced by the bacterial fermentation of dietary fiber. They are reported to have important physiological effects in the intestine. For example, SCFAs influence the functions of the gastrointestinal tract (Ríos-Covián et al., 2016), electrolyte balance (Vidyasagar and Ramakrishna, 2000), and ion transport (Yajima, 1988). The concentration of SCFAs in feces reflects the activity of enteric microorganisms and is important in the relief of constipation by CMB. Our study showed that CMB1 improved the symptoms of constipation and increased the levels of propionic and butyric acids. This increase corresponded to an increase in propionic and butyric acid producing bacteria. Propionic acid producing bacteria are mainly Bacteroidetes, and butyric acid levels are positively correlated with the relative abundance of Firmicutes (Guilloteau et al., 2010). In our study the observed increases in propionic and butyric acid are consistent with an increase in *Prevotella* and *Lactobacillus* in feces samples treated with CMB1, and increase in unclassified S24-7, *Rikenella*, *Bacteroides*, *Dorea*, *Lactobacillus, Dehalobacterium*, *Desulfovibrio, Ruminococcus* and *Coprococcus* in caecal content samples treated with CMB1. It has been widely reported that either *Prevotella* or *Bacteroides* dominates the human gut and they were proposed to be antagonistic (Choi and Chang, 2015). *Prevotella* and *Bacteroides*, which are thought to have had a common ancestor, benefited their host by excluding potential pathogens from colonizing the gut (Ley Ruth, 2016). It also can be seen from the results that the ratio of Firmicutes to Bacteroidetes increased significantly in feces samples of CMB1-treated group (from 0.89 in control group to 1.55 in CMB1group). A higher ratio of Firmicutes to Bacteroidetes is associated with faster transit in the large intestine (Sadik et al., 2004; DelgadoAros et al., 2008). Meanwhile, as observed in this study, the higher abundance of Actinobacteria (*Bifidobacterium*) in feces sample of CMB1-treated group was also correlated with faster colonic transit and in agreement with an earlier study (Parthasarathy et al., 2016). Therefore, it seems that the fecal microbiota profile may be related to colonic transit.

After treatment with CMB1, the genera unclassified S24-7, *Rikenella*, *Bacteroides*, *Dorea*, *Lactobacillus, Dehalobacterium*, *Desulfovibrio, Ruminococcus* and *Coprococcus* increased in caecal content sample. Unclassified S24-7 is a prevalent and abundant bacterial component of the gut microbiome of mammals (Ormerod et al., 2016) and Unclassified S24-7 is more abundant following treatment-induced remission of colitis in mice (Rooks et al., 2014). The genera Firmicutes-*Dorea* and Bacteroidetes-*Bacteroides* were correlated inversely with the production of methane, while the Firmicutes-*Oscillospira* and Bacteroidetes-*odoribacter* were correlated directly with the production of methane (Parthasarathy et al., 2016). In our research, the relative abundances of *Dorea* and *Bacteroides* increased significantly and the genera of *Odoribacter* and *Oscillospira* decreased obviously in caecal microbiota, thus suggest that the caecal microbiota is a better indicator for constipation and methane production than fecal microbiota. Furthermore, the genera Firmicutes-*Coprococcus* and Firmicutes-*Lactobacillu*s which were correlated directly with colonic transit (Parthasarathy et al., 2016), increased significantly in caecum microbiota. These findings suggest that the caecal microbiota is also a better indicator for colonic transit. Taken together, compared with fecal microbiota, cecum microbiota reflects constipation state more comprehensively and accurately.

As mentioned above, adhesive *Bifidobacterium* combination affected fecal and caecal microbiota with different way to non-adherent *Bifidobacterium* combination. Furthermore, changes in the caecal contents microbiota and fecal microbiota were different as well whether treated with adhesive or non-adherent *Bifidobacterium*. It was found that there were seven genera with significant differences between fecal microbiota and caecal content microbiota. Meanwhile, six of these seven genera showed a high abundance in the caecal content compared to the relative abundance in the feces, whether in the CMB1 treatment group or the CMB2 treatment group. Such as the relative abundances of *Oscillibacter*, *Clostridium*, *Anaeroplasma*, *Ruminococcus* and so on. This might be due to the different environments in different part of intestine which resulted in different abundance of intestinal microbes. The intake of *Bifidobacterium* affected the microbiota in these parts of intestine. Due to the differences of microbiota species abundance, the influences by *Bifidobacterium* on caecal contents microbiota and fecal microbiota were different as well. We believe that adhesive *Bifidobacteria* can not only stay in the intestinal tract for a long time, but also produce more metabolites, so as to play a more effective role in changing gut microbiota than that by non-adhesive Bifidobacteria.

In this study, the relative abundances of *Bifidobacterium* (Actinobacteria), *Lactobacillus* (Firmicutes) and *Akkermansia* (Verrucomicrobia), which have been shown to improve the motility of the intestine by provoking the release of 5-HT or by promoting cholinergic pathways (Reigstad et al., 2015), were associated with faster transit in the large intestine. It is also consistent with our previous study (Wang et al., 2017a) where feeding constipated mice with CMB regulated the dysbiosis of the gut microbiota (decreases in the levels of *Bifidobacterium* and *Lactobacillus*) caused by constipation. Recent studies in rodents indicate that *Akkermansia* in the gut might reduce obesity, diabetes and inflammation (Everard et al., 2013). In this study, overgrowth of *Bifidobacterium*, *Lactobacillus* and *Akkermansia* was found to accompany reduction in the number of other bacteria, such as *Anaeroplasma*, *Parabacteroides*, *Clostridium* (in fecal samples) and unclassified Helicobacteraceae (only showed slight downward trend in the caecal contents sample). Some species of *Anaeroplasma*, *Parabacteroides* and *Clostridium* are pathogenic to humans and are associated with colitis and gastroenteritis (Ethan et al., 2011; Michael et al., 2016). Some species of *Helicobacter* (unclassified Helicobacteraceae) are associated with peptic ulcers, chronic gastritis, duodenitis and stomach cancer (Alexander et al., 2014; Watari et al., 2014; Seyed et al., 2015; Sostres et al., 2015). The genera *Clostridium*, *Dorea*, *Oscillibacte*, *Ruminococcus*, *Sporobacter* and *Turicibacter* are considered opportunistic pathogens (Sokol et al., 2008; Jenq et al., 2012; Konikoff and Gophna, 2016). When probiotics are predominated in the gut, opportunistic pathogens can have adjuvant effects, but when pathogenic bacteria are predominated, opportunistic pathogens can have pathogenic effects. In our present study, the overgrowth of probiotics changed the microenvironment of the gut and helped the opportunistic pathogens to develop in the health promoting direction. Thus, the changes in the above genera had a potential regulatory role in relieving constipation. It is certain that the intestinal microbiota showed dysbiosis after constipation. However, whether dysbiosis causes constipation or represents an epiphenomenon remains unclear. Further studies are needed to determine the true cause-and-effect relationship.

## Conclusion

In conclusion, this study demonstrates that strains with adhesion properties (CMB1) can alleviate constipation more efficiently. CMB1 noticeably increased the water content of feces, small intestinal transit rates, the first black stool defecation time and the concentration of propionic and butyric acids. Moreover, constipated mice treated with CMB1 had a unique profile of caecal microbiota that reflect the relief of constipation more comprehensively and accurately, in comparison with fecal microbiota which was only associated with the colonic transit.

## Data Availability

The datasets (gut metagenome Genome sequencing and assembly. SRA accession: PRJNA531550) for this study can be found in the [Sequence Read Archive (SRA)] (https://www.ncbi.nlm.nih.gov/bioproject/PRJNA531550).

## Ethics Statement

This study was approved by the Ethics Committee of the Jiangnan University, China (JN. No. 20150326-0110-21) and performed at the Experimental Animal Center of the Jiangnan University [License No. SYXK(SU)2016-0045].

## Author Contributions

LW and GW conceived and designed the experiments. LW, CC, and SC performed the experiments. LW, GW, Y-kL, and HZ analyzed the data. JZ, HZ, and WC contributed reagents, materials, and analysis tools. LW, GW, and Y-kL wrote the manuscript. All authors contributed to the manuscript revision, and read and approved the submitted version.

## Conflict of Interest Statement

The authors declare that the research was conducted in the absence of any commercial or financial relationships that could be construed as a potential conflict of interest.

## Footnotes

1 http://qiime.sourceforge.net/
