# Supplementary material for: Adhesive Bifidobacterium Induced Changes in Cecal Microbiome Alleviated Constipation in Mice
Source: Front Microbiol. 2019 Aug 13;10:1721. doi: 10.3389/fmicb.2019.01721 (PMC6700325; doi:10.3389/fmicb.2019.01721)
Supplement: Supplementary file 1 [file Data_Sheet_1.docx]

Supplementary Material

**Adhesive *Bifidobacterium* induced changes in cecal microbiome alleviated constipation in mice**

Linlin Wang^1,2,3^, Cailing Chen^1,2^, Shumao Cui^1,2,3,4^, Yuan-kun Lee ^5^, Gang Wang^1,2,3,4*^, Jianxin Zhao^1,2,4^, Hao Zhang^1,2,4,6,7^, Wei Chen^1,2,6,8^

* Corresponding author: Gang Wang

E-mail address: [wanggang@jiangnan.edu.cn](mailto:wanggang@jiangnan.edu.cn)

# Supplementary Figures


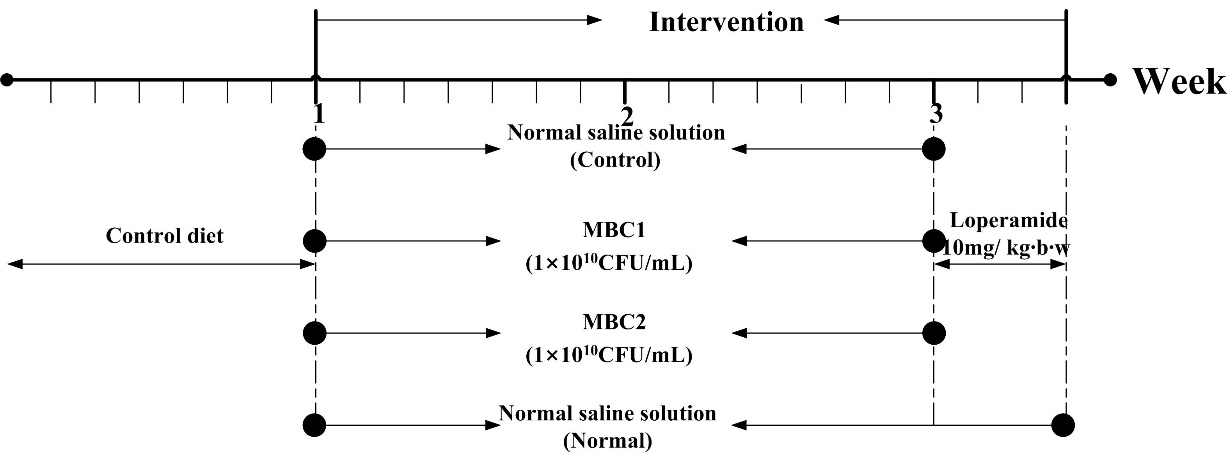


**Supplementary Figure 1.** **Experimental design**

Thirty-two mice were randomly and equally divided into four groups (normal, control, CMB1 and CMB2). In the normal group, mice were gavaged with normal saline solution; in the CMB1 and CMB2 groups, mice were respectively gavaged with 1 × 10^10^ CFU/mL CMB1 and CMB2 for 2 weeks; from day 15 to day 17, all mice were gavaged with loperamide, except the normal group.


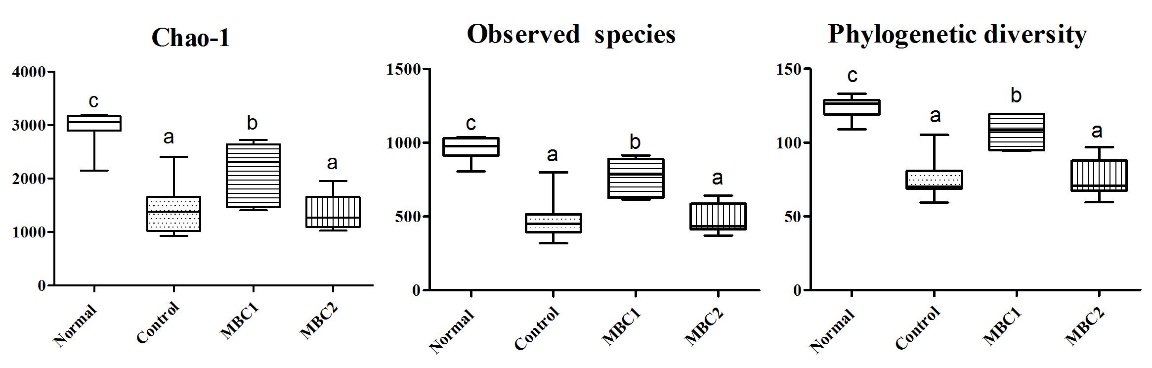


**Supplementary Figure 2.** **Alpha diversity indexes** **of fecal samples in CMB treated mice for 17 days.**

Groups with dissimilar letters differ, *p* < 0.05.


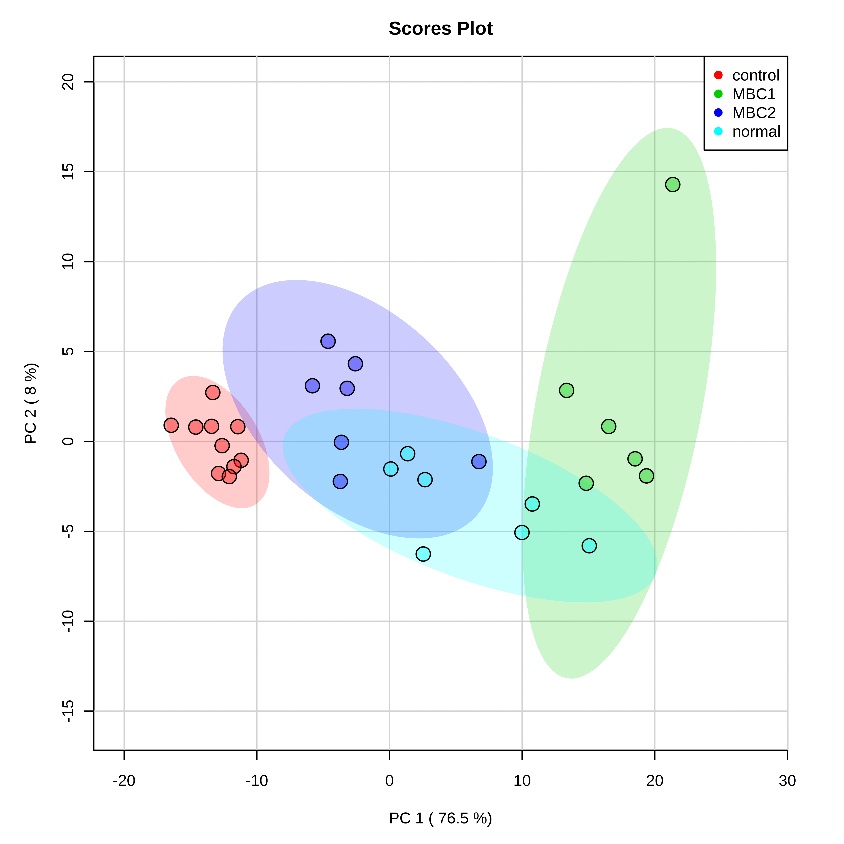


**Supplementary Figure 3. Principal coordinates analysis (PCoA) plots of different treatment groups in faecal groups.**


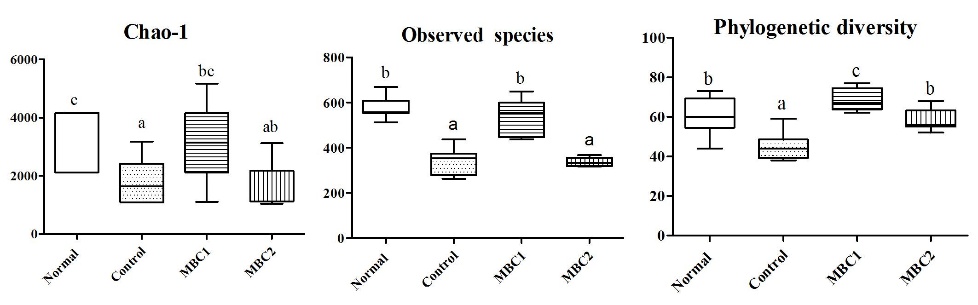


**Supplementary Figure 4. Alpha diversity indexes** **of caecum samples in CMB treated mice for 17 days.**

Groups with dissimilar letters differ, p < 0.05.


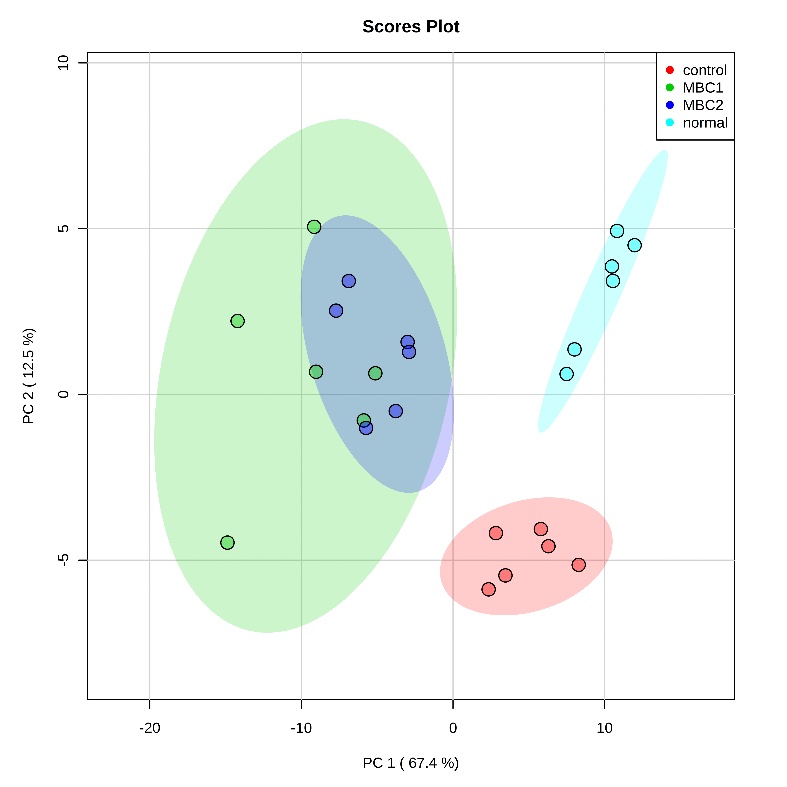


**Supplementary Figure 5. Principal coordinates analysis (PCoA) plots of different treatment groups in caecum samples.**

**
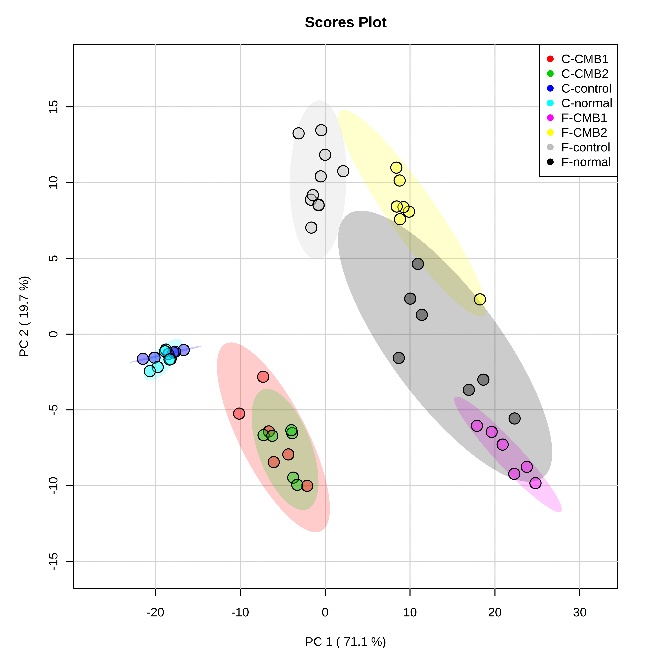
**

**Supplementary Figure 6. Principal coordinates analysis (PCoA) plots of faecal sample compared to caecum content sample.**

.
